# Selectivity of direct plasma treatment and plasma-conditioned media in bone cancer cell lines

**DOI:** 10.1038/s41598-021-96857-9

**Published:** 2021-09-01

**Authors:** Inès Hamouda, Cédric Labay, Uroš Cvelbar, Maria-Pau Ginebra, Cristina Canal

**Affiliations:** 1 Biomaterials, Biomechanics and Tissue Engineering Group, Department of Materials Science and Engineering, and Research Centre for Biomedical Engineering (CREB), Universitat Politècnica de Catalunya (UPC), Av. Eduard Maristany 10-14, 08019 Barcelona, Spain; 2 Barcelona Research Centre in Multiscale Science and Engineering, UPC, Barcelona, Spain; 3 Institut de Recerca Sant Joan de Déu, 08034 Barcelona, Spain; 4 Department of Gaseous Electronics (F-6), Jožef Stefan Institute, Jamova cesta 39, 1000 Ljubljana, Slovenia; 5 Institute for Bioengineering of Catalonia, c/Baldiri i Reixach 10-12, 08028 Barcelona, Spain

**Keywords:** Cancer, Bone cancer, Cancer therapy, Biomedical engineering, Plasma physics

## Abstract

Atmospheric pressure plasma jets have been shown to impact several cancer cell lines, both in vitro and in vivo. These effects are based on the biochemistry of the reactive oxygen and nitrogen species generated by plasmas in physiological liquids, referred to as plasma-conditioned liquids. Plasma-conditioned media are efficient in the generation of reactive species, inducing selective cancer cell death. However, the concentration of reactive species generated by plasma in the cell culture media of different cell types can be highly variable, complicating the ability to draw precise conclusions due to the differential sensitivity of different cells to reactive species. Here, we compared the effects of direct and indirect plasma treatment on non-malignant bone cells (hOBs and hMSCs) and bone cancer cells (SaOs-2s and MG63s) by treating the cells directly or exposing them to previously treated cell culture medium. Biological effects were correlated with the concentrations of reactive species generated in the liquid. A linear increase in reactive species in the cell culture medium was observed with increased plasma treatment time independent of the volume treated. Values up to 700 µM for H_2_O_2_ and 140 µM of NO_2_^−^ were attained in 2 mL after 15 min of plasma treatment in AdvDMEM cell culture media. Selectivity towards bone cancer cells was observed after both direct and indirect plasma treatments, leading to a decrease in bone cancer cell viability at 72 h to 30% for the longest plasma treatment times while maintaining the survival of non-malignant cells. Therefore, plasma-conditioned media may represent the basis for a potentially novel non-invasive technique for bone cancer therapy.

## Introduction

Primary bone cancers, such as osteosarcoma, are developmental diseases that primarily affect children and adolescents. Osteosarcoma is an aggressive malignant neoplasm that arises from primitive transformed cells of mesenchymal origin that exhibit osteoblastic differentiation and produce malignant osteoid. Current therapies for osteosarcoma are not entirely effective, and patients are prone to relapse. In this context, atmospheric pressure plasma jet (APPJ) treatments have arisen as a potential new therapeutic approach for cancer treatment^1–3^. The emerging field of plasma medicine already employs APPJ devices for cancer removal^4,5^. Furthermore, APPJ has the potential to affect cells through complex biochemical processes with the ability to selectively kill cancer cells without affecting non-malignant cells, i.e., the surrounding tissues^6–8^. This effect is primarily attributed to the reactive oxygen and nitrogen species (RONS) generated by APPJ and is responsible for disturbing the cellular metabolic environment^9,10^. However, it is known that other components from plasma, such as electromagnetic fields and visible–UV radiation, as well as thermal heating, can also affect cells. For this reason, many researchers predominantly and preferably rely on direct plasma treatments of cancerogenic cells or tumours.

Promising findings have emerged by targeting cancer cells through their metabolism for oncological therapies^11^. In fact, non-malignant cells already contain a certain level of RONS for their metabolic regulation, while cancer cells exhibit abnormally high levels. The addition of exogenous RONS can surpass the toxicity threshold levels and overwhelm cellular defence mechanisms, leading to apoptotic cell death^12,13^. APPJ-based therapies have already demonstrated selectivity in a variety of cancer cell lines^14–20^, especially direct treatments.

The efficiency of APPJ has been demonstrated in vitro and in vivo by directly treating cells or tumours with plasma jet^16,19^. As mentioned above, direct treatment of cells with APPJ leads to selective cancer cell death without affecting non-malignant cells. As cells are surrounded by biological media, i.e., blood in in vivo or cell culture media in vitro, interactions between APPJ and liquids occur during treatment, leading to biological effects. Based on these findings, plasma-conditioned media were investigated in this work as a potential vehicle of RONS transport, avoiding the effects of visible UV radiation, electric fields and thermal heating from APPJs, which can disturb the cellular environment. The efficiency of the generation of RONS from APPJ depends on the device configuration, the selected parameters (such as carrier gas), the gas flow and the distance of treatment. Furthermore, the chemical composition of the treated liquid, its volume or the cell type employed are important for determining the biological effects of plasmas. It is of great interest to suitably quantify RONS generated in cell culture media under different conditions and their effects on cells to understand the suitable dose of RONS needed to achieve selectivity for cancer cells. As osteosarcoma is difficult to access, a therapy dealing with plasma-conditioned liquids could be adapted to a minimally invasive approach. A few studies have reported direct plasma treatment of three types of osteosarcoma cell lines (U2-OS, MNNG/OS and SaOs-2)^21–23^. Indirect treatment using different cell culture media, in accordance with the cell line employed, was evaluated for its selectivity in the SaOs-2 cell line versus non-malignant cells^24,25^. However, treating different cell culture media resulted in the generation of different amounts of RONS by plasma, which prevented us from drawing definitive conclusions on the sensitivity of the two cell types to the reactive species generated by plasma. Hence, the effect of plasma treatment using the same liquid environment should be assessed. Plasma-conditioned liquids, i.e., saline solutions have also demonstrated selectivity towards SaOs-2, U2-OS and MG63 bone cancer cell lines versus the hMSC cell line^26^. In this work, we aimed to further extend the investigation of direct and indirect APPJ treatment methods. The purpose of this study was to compare the different cytotoxicities of non-malignant bone and bone cancer cell lines. Importantly, to allow a true comparison, the same cell culture medium was employed for all cell types, both during direct plasma treatment or to generate plasma-conditioned media in indirect treatment. The influence of the plasma-treated volume was also evaluated on different bone cells to compare both treatments, ensuring the same conditions for the generation of reactive species. The potential for the use of plasma-conditioned media is studied in this work as a potential tool for future non-invasive therapy for bone cancer.

## Results

### Generation of reactive species by APPJ in the gas phase

**Figure 1:**
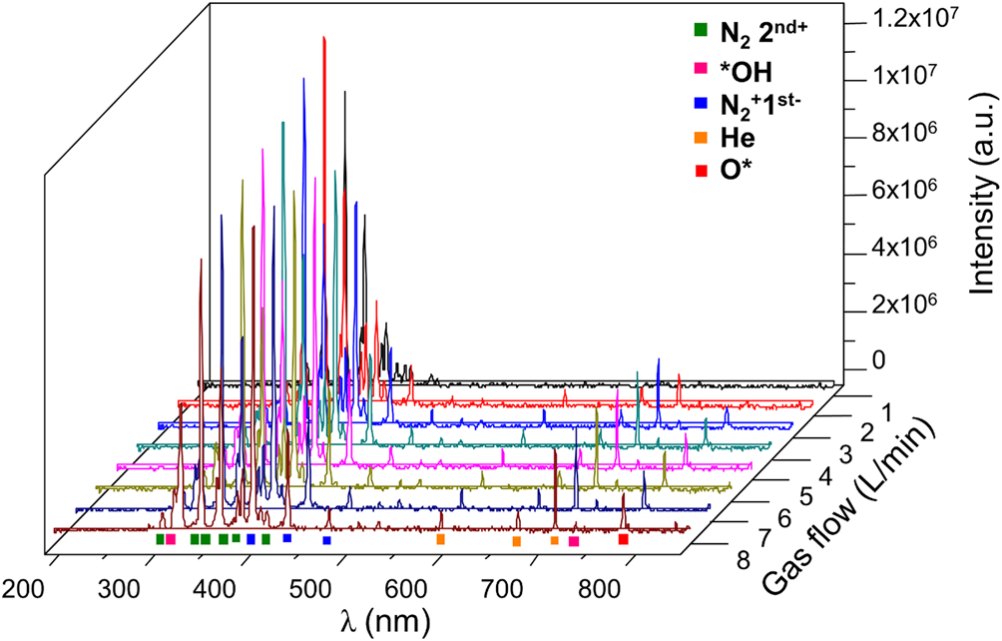
Optical emission spectra of the plasma jet operated with He at different flow rates in ambient air.

The species generated by the APPJ in the gas phase were recorded in air and analysed by optical emission spectroscopy (Fig. 1). Different peaks in emission are attributed to He (carrier gas) (λ = 706 nm) and the surrounding environment O* (λ = 777 nm), *OH (λ = 316 nm), N_2_^+^ 1st positive (λ = 380 nm) and N_2_^+^ 2nd. An increase in the intensity of these peaks was observed with an increase in the gas flow employed up to 6 L/min. The intensity of the spectral lines of these species decreases with the gas flow, as shown in Fig. 1.

### Generation of reactive species in the liquid phase

**Figure 2:**
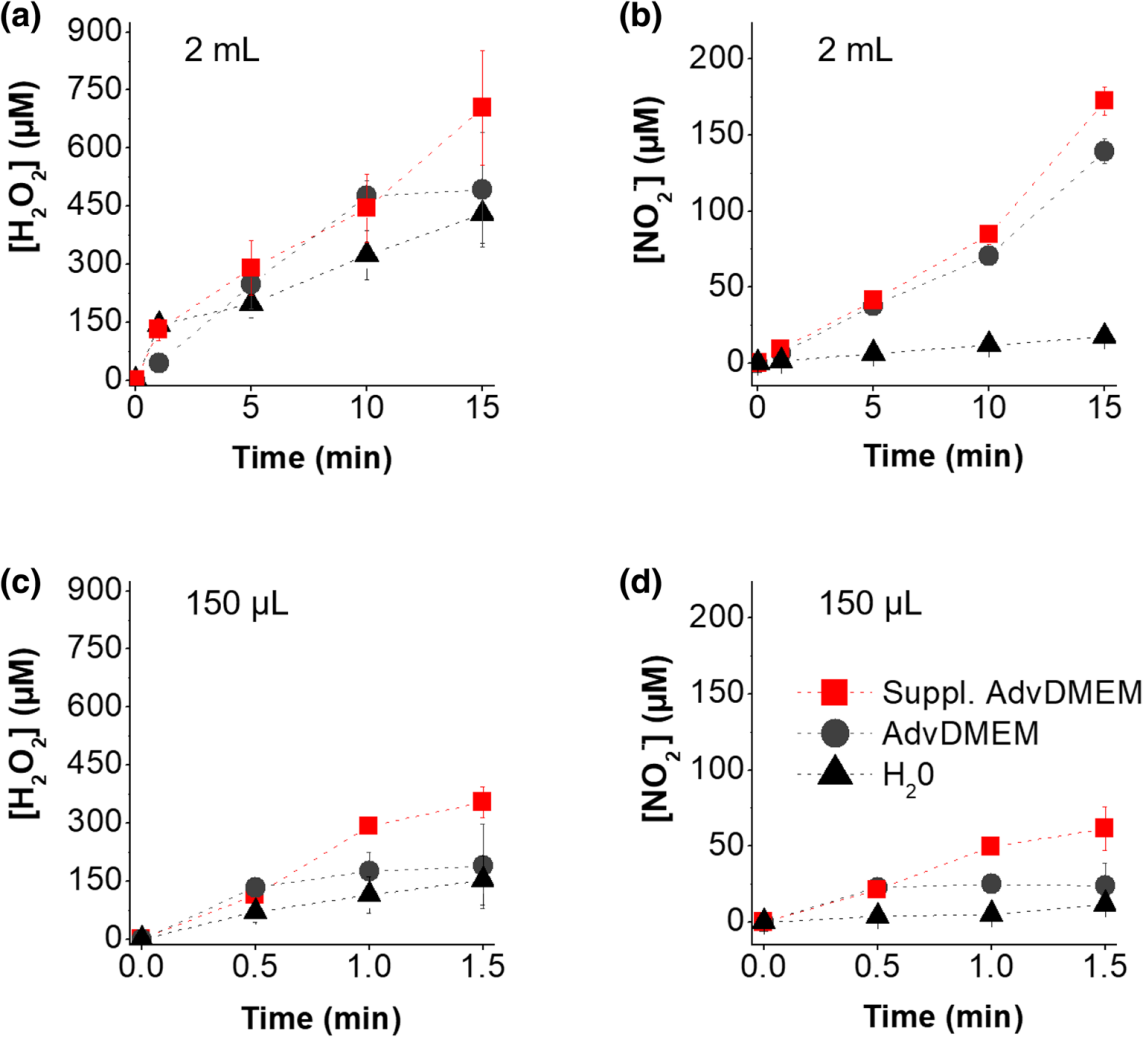
Generation of long-lived reactive species in water (triangle), AdvDMEM (circle) and supplemented AdvDMEM (square) of (**a**) H_2_O_2_ and (**b**) NO_2_^−^ in 2 mL of liquid and of (**c**) H_2_O_2_ and (**d**) NO_2_^−^ in 150 µL of liquid as a function of APPJ treatment time.

The generation of two major reactive species from APPJ was quantified in water, AdvDMEM, and supplemented AdvDMEM, as used for cell culture. The concentrations of two of the main species generated, hydrogen peroxides (H_2_O_2_) and nitrites (NO_2_^−^), were investigated in different volumes following APPJ treatments (Fig. 2). A linear increase in both H_2_O_2_ and NO_2_^−^ following APPJ treatment in all liquids was observed, regardless of the volume treated, as a function of the plasma treatment time. As a general trend, higher concentrations of both H_2_O_2_ and NO_2_^−^ were obtained in the supplemented AdvDMEM with respect to AdvDMEM and water. The concentrations of these generated species highly depend on the APPJ treatment time, volume and chemical composition of the treated liquid. In 2 mL, higher concentrations of H_2_O_2_ and NO_2_^−^ (700 µM and 170 µM, respectively, in supplemented AdvDMEM at 15 min) were reached due to the longer treatment time employed. In a volume of 150 µL, lower concentrations were obtained with shorter treatment times (350 µM and 60 µM H_2_O_2_ and NO_2_^−^, respectively, in supplemented AdvDMEM at 1.5 min). Two different volumes were studied to quantify the species present in the medium during either indirect or direct treatment. For indirect treatment, PCM was generated in 2 mL, from which 150 µL was then transferred into 96-well plates containing the cells for cytotoxicity assays. In contrast, direct treatment was performed with cells seeded into 96-well plates, and thus, the volume of media on the cells was 150 µL.

### Cellular cytotoxicity in response to direct treatment

**Figure 3:**
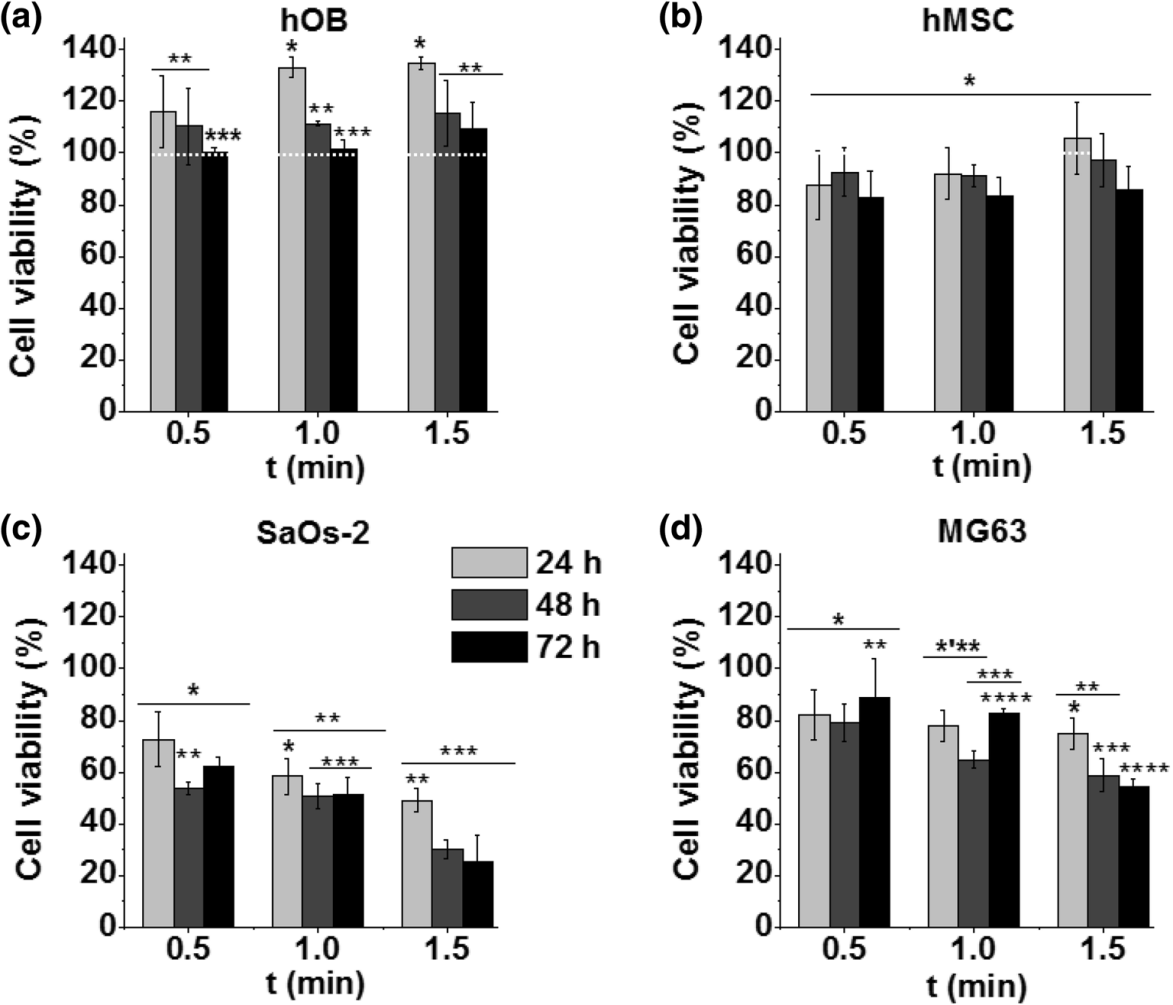
Effects of direct APPJ treatment at different times on the viability of (**a**) hOBs, (**b**) hMSCs, (**c**) SaOs-2s and (**d**) MG63 cells after three different incubation times of 24, 48 and 72 h. The influence of direct APPJ treatment time on cell viability was evaluated for 0.5-, 1- and 1.5-min plasma treatments. Cell viability normalised to control cells (without APPJ treatment). Dashed white line indicates 100% of cell viability with respect to control.

APPJ treatment was directly applied to cells seeded in 96-well plates with 150 µL of fresh supplemented AdvDMEM cell culture media. Two non-malignant primary bone and two osteosarcoma cell lines were compared at different APPJ treatment times. The viability of human osteoblast primary cells (hOBs), human bone marrow-derived mesenchymal stem cells (hMSCs), non-malignant bone cells, sarcoma osteogenic human cells (SaOs-2), and osteosarcoma (MG63) cancer cells after plasma treatment is presented in Fig. 3 at three different incubation times. The results clearly show that both non-malignant cell lines remained fully viable (Fig. 3a,b), as the viability of hMSCs was greater than 80% at all treatment times Furthermore, hOBs exposed to direct plasma initially displayed stimulated cell proliferation with respect to control—which is referenced at 100% cell viability—(Fig. 3a) following APPJ treatment after 24 h of incubation (135 ± 2% for 1.5 min) which was mitigated with incubation time to values close to the control cells (110 ± 10% for 1.5 min at 72 h). hMSCs were more sensitive to direct plasma treatment, exhibiting a small reduction in viability to values between 85 and 100% (Fig. 3b). In osteosarcoma cells, cell viability below 80% was obtained in all cases. Both APPJ treatment time and incubation times decreased the viability of SaOs-2 cells from 73 ± 10% after 0.5 min at 24 h to 25 ± 10% after 1.5 min at 72 h (Fig. 3c). MG63 cells were also less sensitive to plasma treatment, displaying a viability decrease from 75 ± 5% at 24 h to 55 ± 3% after 1.5 min of APPJ treatment at 72 h (Fig. 3d).

### Cellular cytotoxicity in response to indirect treatment

**Figure 4:**
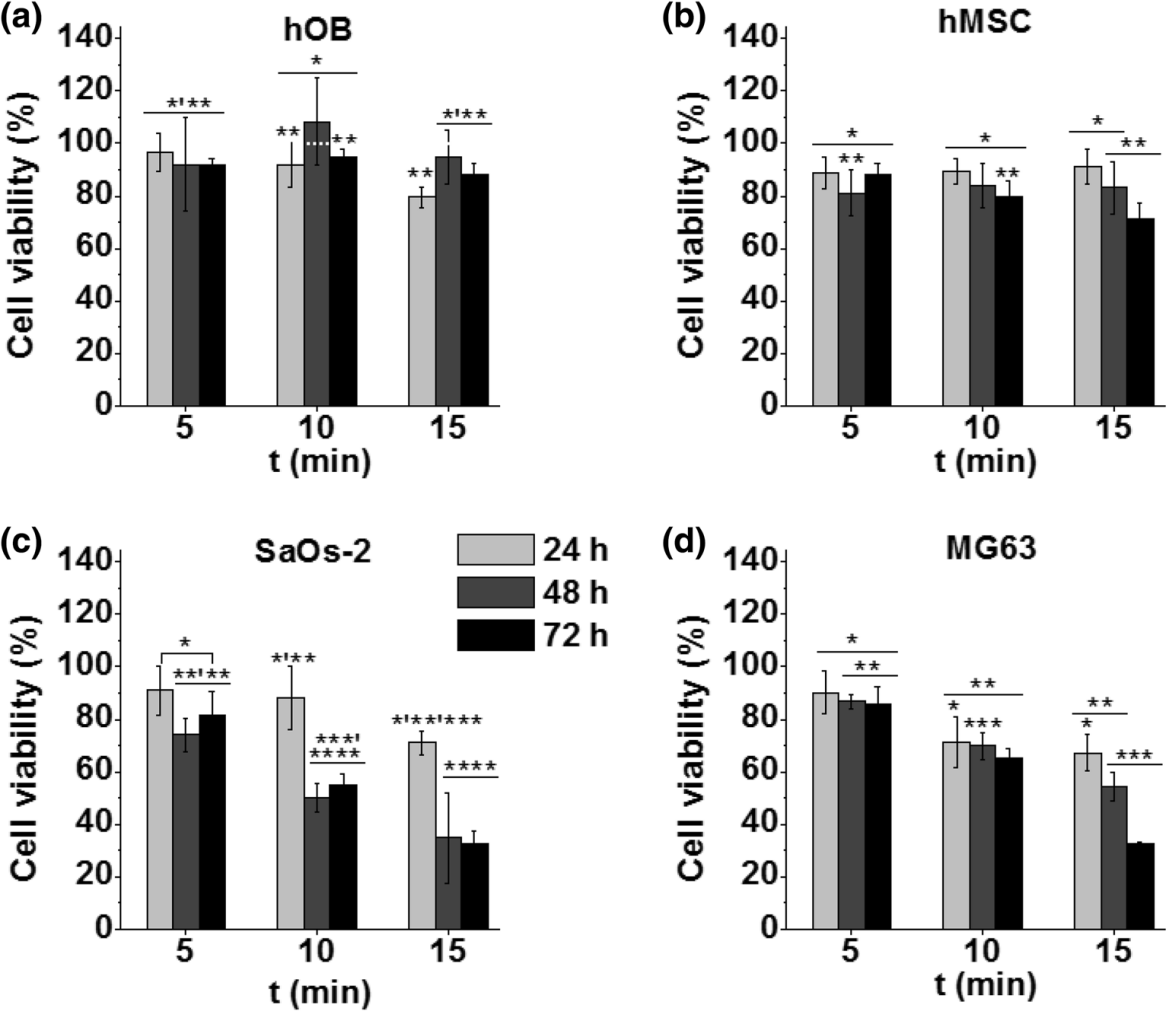
Effects of PCM on the viability of (**a**) hOB, (**b**) hMSC, (**c**) SaOs-2 and (**d**) MG63 cells at three different incubation times: 24, 48 and 72 h. The influence of indirect APPJ treatment time on cell viability was evaluated after 5, 10 and 15 min of plasma treatment. Cell viability normalised to control cells (without PCM). Dashed white line indicates 100% of cell viability with respect to control.

To evaluate indirect treatment, 2 mL of supplemented AdvDMEM was treated between 5 and 15 min with APPJ as described in the experimental section to obtain PCM. Then, 150 µL of this PCM was put in contact with cells seeded under the same conditions as described above. The trends obtained with the indirect treatment (Fig. 4) were similar to those with direct treatment, except for in hOB cells. Indirect treatment with PCM caused a minor reduction in cell viability in both types of non-malignant cells, with cell viability between 80 and 90% (Fig. 4a). A minor decrease in viability was observed in hMSCs treated with 15 min-PCM from 90 ± 10% at 24 h to 70 ± 5% at 72 h (Fig. 4b).

Cell viability in bone cancer cells decreased with increasing plasma treatment time as well as with the incubation time. SaOs-2 cells were more sensitive to plasma treatment, with values of approximately 50% in response to PCM treatment for 10 min or 30% for 15 min-treated PCM (at 72 h of incubation). MG63 cells could initially withstand the effects of PCM fairly well (70% viability with 10 min PCM), but after a certain threshold, i.e., with 15-min PCM, their viability also decreased to 30%.

**Figure 5:**
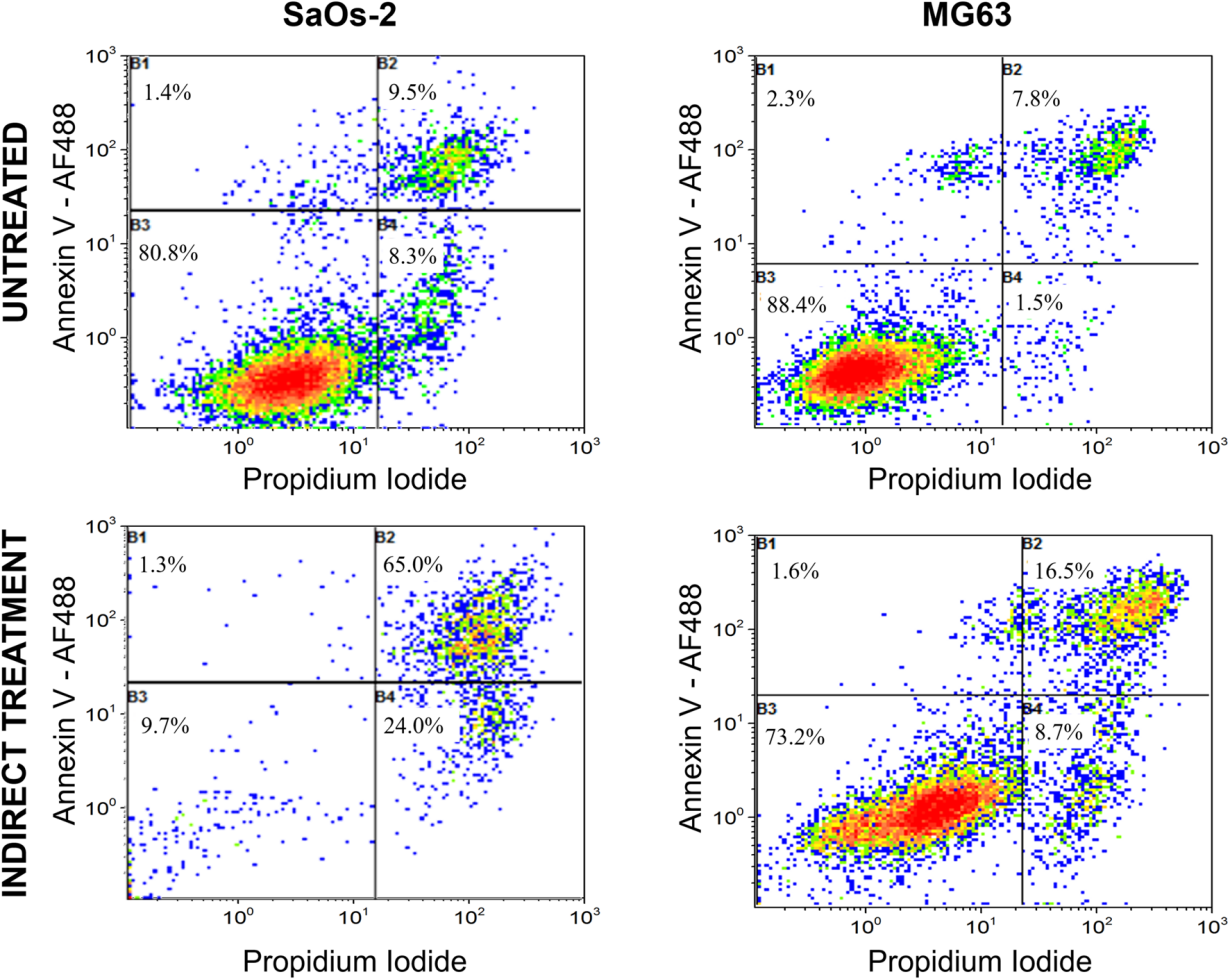
Flow cytometry analysis of SaOs-2 and MG63 cells after 15 min of indirect APPJ treatment and 72 h of incubation.

To discern the mechanism of cancer cell death related to the decrease in cell viability observed in Fig. 4, flow cytometry was performed on SaOs-2 and MG63 osteosarcoma cells (Fig. 5). Indirect plasma treatment of SaOs-2 and MG63 cells with PCM induced apoptosis of bone cancer cells, with all cells in the preapoptotic or apoptotic stage. After 72 h of incubation with 15 min of PCM, 24% of SaOs-2 cells were in the preapoptotic stage and 65% were in the apoptotic stage, compared to 8.3% and 9.5% for the preapoptotic and apoptotic stages of untreated cells, respectively. Similar trends were observed in treated MG63 cells, 6.7% of which were in the preapoptotic stage and 16.5% in the apoptotic stage. For comparison, values were more than threefold lower for the control—in this case, untreated MG63 cells—1.5% and 7.8% for the preapoptotic and apoptotic stages, respectively.

## Discussion

Osteosarcoma (OS) is a primary bone tumour with a low survival rate (between 5 and 60%, depending on the stage of the tumour at diagnosis), so alternative strategies to the current surgery and chemotherapy treatments are urgently needed. Recent studies^21,24,27–30^ have already investigated the direct effects of cold atmospheric pressure plasma jets in OS cells (essentially U2-OS and MNNG/OS), reporting a decrease in OS cell proliferation. While in general, it is claimed that cold plasmas are selective and do not affect non-malignant cells, many works focus on investigating the effects of plasmas on cancer cells, while non-malignant cells are often disregarded.

For this reason, the effects of helium APPJ on cytotoxicity (Figs. 3 and 4) and cell death mechanism (Fig. 5) were investigated here in two OS cell lines (SaOs-2 and MG63) and two non-malignant primary cell lines (hMSCs and hOBs). The obtained results indicate that the proposed direct APPJ treatment is selective, allowing survival of the non-malignant cells with cell viability of 100% or higher (Fig. 3a,b), while reflecting a progressive decrease in cell viability for cancer cells (down to 30% for SaOs-2 cells and 55% for MG63 after just 1.5 min of treatment—Fig. 3c,d). This confirms the findings for other cancer types in previous studies^31^ where, as here (Fig. 5), indirect treatment with plasma conditioned medium was found to lead to DNA damage and apoptosis preferably in osteosarcoma cells, while preventing apoptosis in non-malignant hBM-MSCs^25^. This holds great promise for a future clinical scenario as this might avoid the undesirable side effects of current therapies (i.e. chemotherapy).

Moreover, enhanced cell death in SaOs-2 and MG63 cells was recorded with increasing APPJ treatment time, and these effects were potentiated with increased incubation time, which is in agreement with previously obtained results^24,25^.

**Figure 6:**
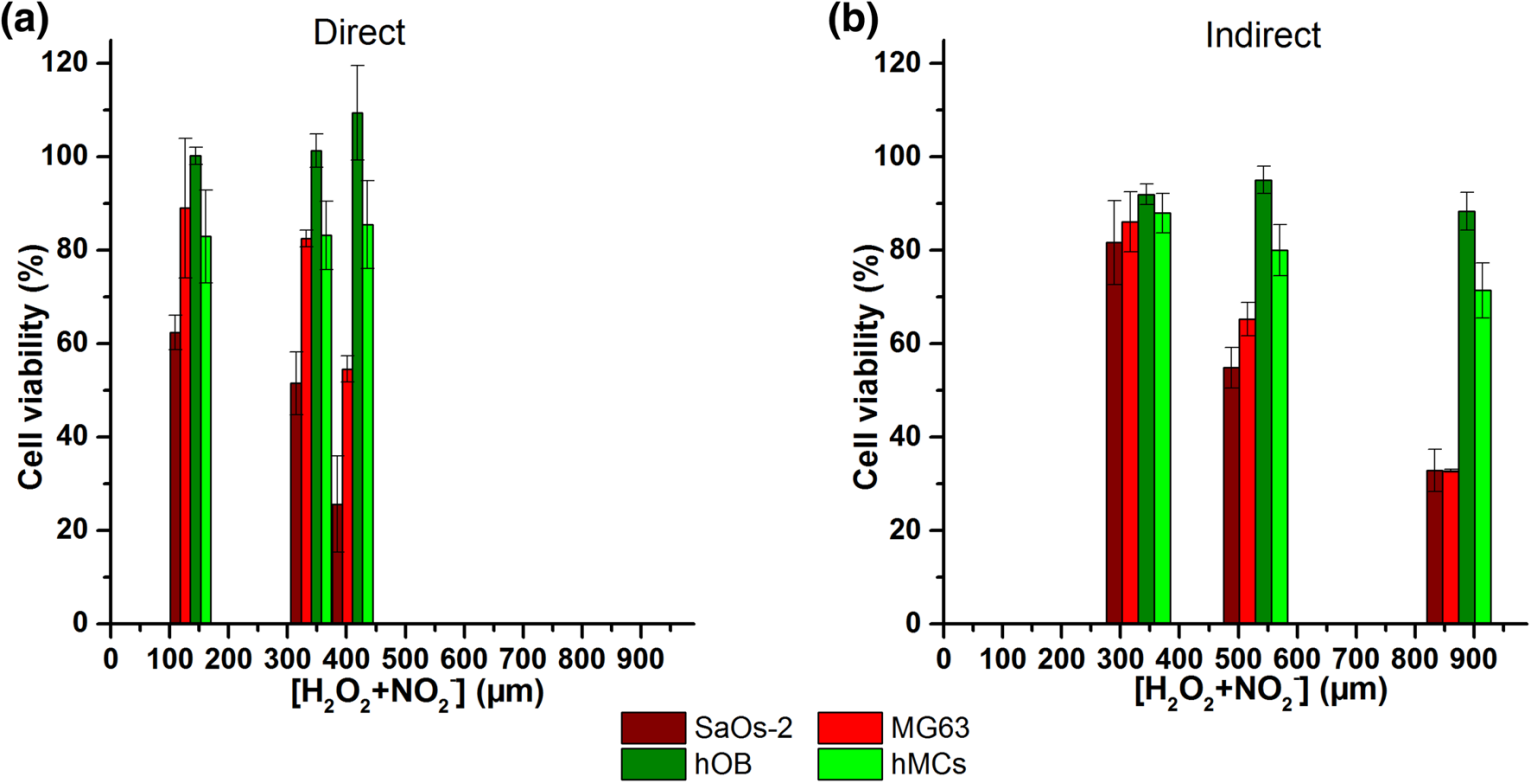
Relationship between doses of long-lived reactive species [H_2_O_2_ + NO_2_^−^] delivered and cell viability of SaOs-2, MG63, hOB and hMSCs at 72 h for direct APPJ treatment (**a**) and PCM (**b**).

Since the bone is an organ that requires surgery to be accessed by APPJ, we also tried to take advantage of the similar cell cytotoxic effects attributed to plasma-conditioned liquids^9^. Such an approach could be interesting in view of minimally invasive therapy, where a plasma-conditioned liquid can be injected into the tumour site. Because of this, the effects of direct APPJ treatment (Fig. 3) were compared to plasma-conditioned media (PCM) (Fig. 4). PCM was obtained from the treatment of cell culture media with APPJ and then transferred onto the seeded cells. While globally similar effects (cytotoxicity and selectivity) as those observed with the direct plasma treatment were observed, it is true that longer treatment times are needed to obtain equivalent effects with PCM (Fig. 6). This indicates the involvement and enhancement of biological activity by plasma-related effects, such as UV and electromagnetic fields. Moreover, the chemistry of the plasma-conditioned liquid is of critical importance^32–34^, as it determines the type and concentration of reactive species. Under normal conditions of cell culture, each cell line is grown in its particular cell culture medium, so some of the research done earlier used different PCMs for each cell line, which hampered comparisons^24^.

Here, supplemented AdvDMEM was employed (composition detailed in Supplementary Materials—Table S1), which includes proteins, amino acids, sugars, salts, etc. The wide variety of molecules in the media justifies the higher concentration of RONS generated by the APPJ compared to water (Fig. 2). Moreover, the higher concentration of RONS in the supplemented advDMEM as compared to its unsupplemented counterpart can be explained by the protein content introduced by the 10% FBS. In a recent paper of Ranieri et al.^35^, the authors also found significantly higher concentrations of peroxides in supplemented medium than in water. In principle, the organic and oxidizable components of the culture medium, as well as the higher pH, should buffer the oxidative capacity of the plasma-generated RONS and reduce their concentrations. However, the higher concentrations observed in the medium suggest the formation of organic peroxides from plasma sources. This was also previously hypothesised by Privat-Maldonado et al.^36^, based on the reaction between solvated ozone (O_3_) and organic compounds to produce higher concentrations of H_2_O_2_ in comparison to water. This is also consistent with our findings^37^ where gelatin solutions treated with plasma jets lead to higher amounts of reactive species.

As observed in other works, the concentration of peroxides generated by this APPJ was higher than that of nitrites. However, due to the presence of pyruvate, a scavenger of H_2_O_2,_ in this cell culture medium, the concentration here was lower than that previously reported^25^. Plasma treatment of liquids typically leads to important acidification (i.e. down to pH = 2 in water^37^). For the cell culture media used in the present experiments, the pH of the PCM only decreased by less than 0.5 in 15 min as a result of the buffering effect of the proteins and amino acids present in the cell culture media, so this is not expected to influence cell viability.

Surprisingly, the cell viability values obtained in our research in response to direct APPJ treatment (Fig. 3) and indirect treatment with PCM (Fig. 4) were very similar (Table 1) (i.e. for SaOs-2 at 72 h, and the longest time was approximately 25–30% with both treatments; for MG63, the longest time was 55% for the direct treatment and 33% for the indirect treatment. This result indicates the capabilities of both approaches to treatment therapies.

**Table 1:** Compilation of the concentrations of H_2_O_2_ and NO_2_^−^ for the corresponding cell viability in SaOs-2 and MG63 cells at the three treatment times investigated for either direct APPJ treatment or indirect treatment with PCM.

	Direct treatment				PCM			
	[RONS] (µM)		Cell viability (%) *		[RONS] (µM)		Cell viability (%) *	
	H_2_O_2_	NO_2_^−^	SaOs-2	MG63	H_2_O_2_	NO_2_^−^	SaOs-2	MG63
T1	114 ± 37	21 ± 5	62 ± 4	89 ± 15	290 ± 70	40 ± 5	82 ± 5	86 ± 6
T2	290 ± 6	50 ± 1	52 ± 7	83 ± 2	444 ± 90	85 ± 6	55 ± 4	62 ± 4
T3	350 ± 40	60 ± 14	26 ± 10	55 ± 3	704 ± 150	170 ± 9	33 ± 5	33 ± 0.3

T1 = 0.5 min for direct, or 5 min for PCM. T2 = 1 min for direct, or 10 min for PCM. T3 = 1.5 min for direct, or 15 min for PCM.

*Cell viability at 72 h of incubation.

When considering the direct effects from APPJ, it must be remarked that the APPJ employed remains at near-ambient temperature and that the gas flow leads to liquid evaporation during treatment, which can be associated to a certain cooling of the liquid (Fig. S1). Moreover, direct treatment with APPJ contains, in addition to the RONS already discussed, a number of additional stimuli that can affect the cells, namely, UV–Vis and electromagnetic radiation, photons, electrons, etc. However, analysis of the concentration of RONS in both treatments (Table 1) shows that the concentrations of H_2_O_2_ and NO_2_^−^ are nearly doubled for the PCM employed in the indirect treatment with respect to the direct APPJ treatment. This difference in concentration is due to the much longer treatment times employed during the treatment/preparation of the larger volume required for the indirect treatment (as shown in Table 1). Thus, despite that lower concentrations of RONS are generated here in the direct treatment conditions, the similar cell viability values recorded between direct treatment and PCM indicate that the effects of the former must be ascribed not only to RONS but also to the physical stimuli from APPJ affecting the cells during direct treatment (i.e. electromagnetic fields). It has been reported that some effects of direct plasma treatments are similar to those produced by physical therapies like electroporation^38^, leading for instance to membrane permeabilization^28^. Other effects reported by direct plasma treatment of relevance for cancer are a decreased cell migration velocity^17^ or stimulating the immune system^39^.

Finally, our research demonstrated that the higher concentrations of RONS generated with longer APPJ treatment times in the PCM provide nearly equivalent biological results as direct APPJ treatment (Fig. 6), triggering cell death by apoptotic mechanisms, in agreement with previous studies^40–45^. Here, there is an interplay in the role of important RONS such as H_2_O_2_ or NO_2_^−^, which supply a promising tool for direct treatment or an interesting alternative for the treatment of bone tumours using a minimally invasive approach employing plasma-conditioned liquids, especially in larger volumes prepared over longer periods. It has been shown^25^ that equilibrated cocktails of RONS are needed to maintain selectivity of plasma conditioned media towards non-malignant cells, and that overwhelming concentrations of H_2_O_2_ eliminate this selectivity. It is often speculated whether the RONS generated by plasmas could be added artificially to produce the same effects, and while this can be true, for instance by adding H_2_O_2_ in vitro in 2D monolayer cultures, it has been demonstrated that in relevant tumor models (i.e. ex vivo organotypic cultures), peroxides have no effect on tumor cells^26^, indicating that it is the complex cocktail of RONS generated in PCM that is needed for a potential therapy.

Considering that cell culture medium is not clinically approved, using saline solutions to obtain plasma conditioned liquids is a promising option for clinical translation. Relevant works have reported the successful anticancer capacity of saline solutions as a plasma-conditioned liquid both in vitro and in vivo^46–50^, even in osteosarcoma^26^, and plasma-conditioned liquid saline solutions, such as PBS, NaCl 0.9% and Ringer’s lactate, are already being investigated in vivo^51^.

Herein, APPJ treatments were explored as a potential alternative to current bone cancer treatments. The anticancer capacity of direct APPJ treatment and indirect treatment with plasma-conditioned media are both effective in vitro and diminish the viability of cancer cells. The viability of cancer cells decreased from 80 to 30% with treatment time and incubation after treatment, while non-malignant cells maintained their viability at levels above than 80%. The effective selectivity of bone cancer cell lines versus non-malignant cell lines is dependent on the dose of reactive species generated in the media. Under our experimental conditions, higher concentrations of H_2_O_2_ and NO_2_^−^ are generated in larger cell culture volumes due to the longer APPJ preparation times, leading to similar biological results as the direct treatment, which in addition to the reactive species, involves other effects, such as UV–Vis radiation, a strong electromagnetic field, and local cooling due to evaporation (Fig. S1). Therefore, employing plasma-conditioned liquids could provide a promising alternative for minimally invasive bone cancer therapy, which is based on tailoring RONS species in liquid and has many equivalent effects to direct plasma treatments of tumours.

## Methods

### Materials

Sarcoma osteogenic cells (SaOs-2, ATCC, USA), passages 10–24, and human osteosarcoma cells (MG63, CRL-1427, ATCC, USA), passages 20–34, were used as cancer cell lines. Human osteoblast primary cells (hOB, 406-05A, Sigma-Aldrich, USA), passages 3–4, and human bone marrow-derived mesenchymal stem cells (hMSCs; Tebu-bio, France) from passage four were used as primary non-malignant cell lines. For in vitro cell culture, Advanced Dulbecco’s Modified Eagle Medium (AdvDMEM), Dulbecco’s Modified Eagle Medium (DMEM), Osteoblast Growth Medium (ready to use), Annexin V and propidium iodide were purchased from Gibco Life Technologies (Thermo Fisher Scientific). McCoy’s 5A culture medium was purchased from Sigma-Aldrich. Mammalian Protein Extraction Reagent (M-PER) was purchased from Thermo Scientific. Foetal bovine serum (FBS), L-glutamine, penicillin, streptomycin, sodium pyruvate 100x, trypsin (TrypLE) and Alexa Fluor 488 (AF 488) were purchased from Invitrogen. Lactate dehydrogenase activity (LDH, Cytotoxicity Detection Kit) was purchased from Roche Applied Science. SILACTM AdvDMEM (without phenol red) used for RONS detection in cell culture media was purchased from Gibco Life Technologies. Sulphanilamide (purity ≥ 99%, M.W.: 172.20 g/mol, powder form), N-(1-naphthyl) ethylenediamine (purity > 98%, M.W.: 172.20 g/mol; powder form), sodium nitrite (NaNO_2_) (purity of 99.999%, M. W: 69.00 g/mol; powder form), Amplex™ Red reagent (M.W.: 257.25 g/mol, powder form), horseradish peroxidase enzyme type VI (HRP) and hydrogen peroxide solution (30% w/w in H_2_O, M.W.: 34.01 g/mol; liquid form) were purchased from Sigma-Aldrich. Phosphate-buffered saline tablets (PBS) were purchased from Thermo Fisher Scientific. All reagents used for reactive species detection were prepared using Milli-Q water (Millipore, Merck). All reagents were used as received in their chemical grade. Helium (He 5.0) for plasma treatments was supplied by PRAXAIR, Spain.

### Plasma treatment of liquid media

**Figure 7:**
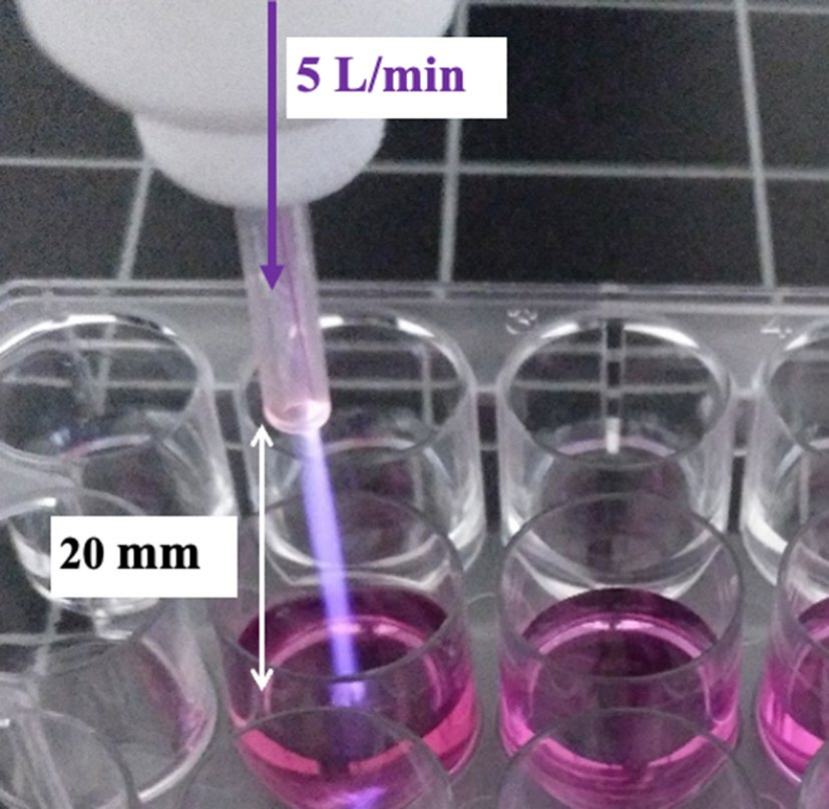
Experimental setup of APPJ treatment of 2 mL of cell culture media to obtain PCM.

Plasma-conditioned media (PCM) was obtained by treating supplemented AdvDMEM cell culture media—or water as a control—using an atmospheric pressure plasma jet (APPJ) made of a single ungrounded electrode, as previously described^52^. The discharge electrode was a copper wire with a diameter of 0.1 mm inserted inside a 1.2 mm inner diameter quartz capillary tube covered by a polytetrafluoroethylene (PTFE) holder. The electrode was connected to a commercial high voltage power supply from Conrad Electronics (nominally 6 W power consumption). The discharge was operated with a sinusoidal waveform at 25 kHz with (U) ~ 2 kV and (I) ~ 3 mA. Helium flow was used as a plasma carrier gas and controlled at 5 L/min by a Bronkhorst MassView flow controller (Bronkhorst, Netherlands). The working distance between the capillary of the plasma device and the liquid medium surface was fixed at 20 mm (Fig. 7). Two different volumes of liquids were investigated: a volume of 2 mL treated for 5, 10 and 15 min in 24-well plates for indirect treatment and a volume of 150 μL treated for 0.5, 1.0 and 1.5 min in 96-well plates (Nunclon Delta Surface, Thermo Fisher Scientific).

### Optical emission spectroscopy

Optical emission spectroscopy (OES) was employed to determine the primary optically emitting species of plasma. The equipment used was an F600-UVVIS-SR spectrometer (StellarNet Inc., USA), which was connected to an Ocean Insight QP600-2-SR optical fibre (Ocean Insight, USA) with a lens collecting information from the measuring point near the plasma jet (10 mm). The influence of gas flow between 0 and 8 L/min was evaluated with respect to the generation of reactive species in the gas phase. All results were obtained using an integration time of 1000 ms and an average of 10 scans. Data were processed using SpectraWiz software (StellarNet Inc., USA).

### Detection of reactive species

Reactive species were quantified in water as a control liquid, in SILAC™ AdvDMEM, and in supplemented SILAC™ AdvDMEM (both without Phenol Red) cell culture media following APPJ treatment in different volumes as previously described.

The concentration of nitrites (NO_2_^−^) was determined using the Griess reaction method^53–55^. Griess reagent was obtained by dissolving 1% wt/v sulphanilamide, 0.1% wt/v N-(1-naphtyl) ethylenediamine and 5% wt/v phosphoric acid in Milli-Q water. Fifty microlitres of Griess reagent was added to 50 μL of plasma-treated samples in 96-well plates. The plates were incubated for 10 min at room temperature protected from light. The absorbance was measured at λ_abs_ = 540 nm using a Synergy Hybrid Multi-Mode Microplate Reader (Biotek, USA). The calibration curve of [NO_2_^−^] was prepared by diluting NaNO_2_ into the corresponding medium. Similarly, the concentration of hydrogen peroxides (H_2_O_2_) was evaluated by fluorescence spectroscopy using Amplex™ Red Reagent (AR) and horseradish peroxidase (HRP) enzyme, following the supplier’s protocol. H_2_O_2_ generation with APPJ was measured through the detection of resorufin, a fluorescent compound, the final product of the reaction of H_2_O_2_ with AR, using HRP as a reaction catalyst^56^. The samples were incubated in the dark at 37 °C for 30 min. Fluorescence intensity was measured using a Synergy Hybrid Multi-Mode Microplate Reader (Biotek, USA) with excitation and emission wavelengths of 560/20 nm and 590/20 nm, respectively. A calibration curve was prepared using a 30% wt/wt H_2_O hydrogen peroxide solution in the corresponding medium.

### In vitro cell experiments

#### Cell culture

McCoy’s 5A was used for the growth of SaOs-2 cells, DMEM for MG63 cells and AdvDMEM for hMSCs, all supplemented with 10% FBS, 1% l-glutamine and 1% penicillin (50 U/mL)/streptomycin (50 μg/mg) (P/S). Sodium pyruvate (1%) was also added to McCoy’s 5A culture medium. Osteoblast growth medium supplemented with 1% P/S was used for hOB cell culture. Each cell type was cultured in its own cell culture media. All plasma treatments were performed in supplemented AdvDMEM with 10% FBS, 1% L-glutamine and 1% P/S. In all experiments, cell culture media in contact with cells was replaced with fresh supplemented AdvDMEM before plasma treatment or with plasma-treated supplemented AdvDMEM.

#### Direct treatment

For direct treatment, subconfluent cells were detached from the flask using trypsin, centrifuged and seeded at a density of 1 × 10^4^ cells per well in 96-well plates with 150 µL of their corresponding complete cell medium. After 24 h of incubation (37 °C, 95% humidity, 5% CO_2_) to allow cell adhesion, the cell culture media was replaced with fresh supplemented AdvDMEM before plasma treatment. APPJ treatment was subsequently performed on the wells containing the adhered cells covered by the cell culture media, according to the plasma treatment conditions described previously (20 mm distance and 5 L/min gas flow). The influence of plasma treatment time on cell viability was evaluated for direct treatment from 0.5 to 1.5 min and for incubation times from 24 to 72 h.

#### Indirect treatment

Indirect treatment refers to replacing cell culture medium with PCM in the seeded cells. Briefly, 1 × 10^4^ cells were seeded into 96-well plates with 150 µL of their corresponding media and allowed to adhere for 24 h. Then, 2 mL of fresh supplemented AdvDMEM cell culture media was treated with APPJ to obtain PCM. Cell culture medium was immediately replaced with 150 µL of this PCM in the wells containing the adherent cells. The influence of plasma treatment time on cell viability was evaluated for indirect treatment from 5 to 15 min. The control refers to untreated cells, where untreated fresh AdvDMEM media was used to replace the cell culture media. Plates were incubated at 37 °C under a 5% CO_2_ and 95% humidity atmosphere for 24 h, 48 h and 72 h for further evaluation of cell viability.

#### Cell viability

After each incubation period, cells were lysed with 100 μL of M-PER. The lysates were analysed to quantify the number of cells by measuring LDH following the manufacturer’s protocols. This allows quantification and measurement of the number of cells. For both direct and indirect treatments, a negative control with no cells and only untreated culture medium as well as a positive control with the corresponding cell type in the untreated medium were evaluated. This positive control was employed as a reference for 100% cell viability. Absorbance was measured at λ = 492 nm using a Synergy HTX multimode microplate reader (BioTek, USA), and cell viability was normalized to cells only.

#### Flow cytometry

The cell death mechanism of SaOs-2 and MG63 cells was analysed using flow cytometry, which was performed after indirect cell treatment. A total of 8 × 10^4^ cells were seeded into 24-well plates with 1.2 mL of their corresponding media and incubated for 24 h for adhesion. Then, 2 mL of fresh supplemented AdvDMEM cell culture media was treated with APPJ for 15 min under the conditions described in “Generation of reactive species in the liquid phase”. Cell culture medium was then replaced with 1.2 mL of this PCM in the wells containing the adhered cells. After 72 h of incubation, the supernatants were collected, and the cells were detached using trypsin and centrifuged. Afterwards, the collected cells were stained for a biochemical marker of apoptosis, Alexa Fluor 488 (AF 488), Annexin V, and a marker of cell membrane integrity, propidium iodide (PI), following the supplier’s instructions (Vybrant apoptosis assay kit, Molecular probes). The annexin V marker has good affinity for phosphatidylserine (PS), which is located on the cytoplasmic surface membrane of normal viable cells. When a cell is in an apoptotic state, PS is translocated from the inner to the outer leaflet of the plasma membrane, exposing PS to the external environment. PI is a marker of necrosis, as it is impermeant to live and apoptotic cells. It penetrates the damaged membranes of necrotic cells, binding tightly to the nucleic acids in the cell, which stain for red fluorescence. Apoptotic, necrotic and non-malignant cell states were analysed in a Gallios multi-colour flow cytometer instrument (Beckman Coulter, Inc., Fullerton, CA) set up with the 3-laser ten colour standard configuration. Annexin V-AF488 and PI were selectively excited at 488 nm. Forward scatter, side scatters, green fluorescence (525/40 nm) from Annexin V-AF 488 and red fluorescence (695/30 nm) emitted by PI were collected simultaneously using logarithmic scales. Forward scatter was used as a discriminating parameter.

#### Statistics

The results presented are the average of data from three independent experimental replicates. Statistical differences were determined using one-way ANOVA with Tukey’s post hoc tests using Minitab 18 software (Minitab Inc., USA). Statistical significance was considered when p < 0.05. Data are presented as the mean ± standard deviation.

## Supplementary Information

Supplementary Information.

## References

[1] Weltmann K-D (2012). Plasma processes and plasma sources in medicine. Contrib. Plasma Phys..

[2] Park GY (2012). Atmospheric-pressure plasma sources for biomedical applications. Plasma Sources Sci. Technol..

[3] Kajiyama H (2017). Future perspective of strategic non-thermal plasma therapy for cancer treatment. J. Clin. Biochem. Nutr..

[4] Laroussi M (2018). Plasma medicine: A brief introduction. Plasma.

[5] Tanaka H (2017). State of the art in medical applications using non-thermal atmospheric pressure plasma. Rev. Mod. Plasma Phys..

[6] Bauer G, Graves DB (2016). Mechanisms of selective antitumor action of cold atmospheric plasma-derived reactive oxygen and nitrogen species. Plasma Process. Polym..

[7] Laroussi M (2015). Low-temperature plasma jet for biomedical applications: A review. IEEE Trans. Plasma Sci..

[8] Graves DB (2014). Low temperature plasma biomedicine: A tutorial review. Phys. Plasmas.

[9] Graves DB (2014). Reactive species from cold atmospheric plasma: Implications for cancer therapy. Plasma Process. Polym..

[10] Volotskova O, Hawley TS, Stepp MA, Keidar M (2012). Targeting the cancer cell cycle by cold atmospheric plasma. Sci. Rep..

[11] Fruehauf JP, Meyskens FL (2007). Reactive oxygen species: A breath of life or death?. Clin. Cancer Res..

[12] Trachootham D, Alexandre J, Huang P (2009). Targeting cancer cells by ROS-mediated mechanisms: A radical therapeutic approach?. Nat. Rev. Drug Discov..

[13] Schumacker PT (2006). Reactive oxygen species in cancer cells: Live by the sword, die by the sword. Cancer Cell.

[14] Ratovitski EA (2014). Anti-cancer therapies of 21st century: Novel approach to treat human cancers using cold atmospheric plasma. Plasma Process. Polym..

[15] Hirst AM, Frame FM, Arya M, Maitland NJ, O’Connell D (2016). Low temperature plasmas as emerging cancer therapeutics: The state of play and thoughts for the future. Tumor Biol..

[16] Keidar M (2013). Cold atmospheric plasma in cancer therapy. Phys. Plasmas.

[17] Keidar M (2015). Plasma for cancer treatment. Plasma Sources Sci. Technol..

[18] Keidar M, Yan D, Beilis II, Trink B, Sherman JH (2018). Plasmas for treating cancer: Opportunities for adaptive and self-adaptive approaches. Trends Biotechnol..

[19] Yan D, Sherman JH, Keidar M (2017). Cold atmospheric plasma, a novel promising anti-cancer treatment modality. Oncotarget.

[20] Keidar M (2011). Cold plasma selectivity and the possibility of a paradigm shift in cancer therapy. Br. J. Cancer.

[21] Gümbel D (2016). New treatment options for osteosarcoma: Inactivation of osteosarcoma cells by cold atmospheric plasma. Anticancer Res..

[22] Gümbel D (2017). Cold atmospheric plasma in the treatment of osteosarcoma. Int. J. Mol. Sci..

[23] Gumbel D (2017). Peroxiredoxin expression of human osteosarcoma cells is influenced by cold atmospheric plasma treatment. Anticancer Res..

[24] Canal C (2017). Plasma-induced selectivity in bone cancer cells death. Free Radic. Biol. Med..

[25] Tornin J (2019). Pyruvate plays a main role in the antitumoral selectivity of cold atmospheric plasma in osteosarcoma. Sci. Rep..

[26] Mateu-Sanz M (2020). Cold plasma-treated ringer’s saline: A weapon to target osteosarcoma. Cancers.

[27] Jacoby JM (2020). An innovative therapeutic option for the treatment of skeletal sarcomas: Elimination of osteo-and ewing’s sarcoma cells using physical gas plasma. Int. J. Mol. Sci. Artic. Int. J. Mol. Sci..

[28] Haralambiev L, Nitsch A, Muzzio D (2020). The effect of cold atmospheric plasma on the membrane permeability of human osteosarcoma cells. Anticancer Res..

[29] Haralambiev L, Wien L, Gelbrich N, Kramer A (2019). Effects of cold atmospheric plasma on the expression of chemokines, growth factors, TNF superfamily members, interleukins, and cytokines in human osteosarcoma cells. Anticancer Res..

[30] Gumbel D (2017). Comparison of cold atmospheric plasma Devices’ efficacy on osteosarcoma and fibroblastic in vitro cell models. Anticancer Res..

[31] Dubuc A (2018). Use of cold-atmospheric plasma in oncology: A concise systematic review. Ther. Adv. Med. Oncol..

[32] Khlyustova A, Labay C, Machala Z, Ginebra M-P, Canal C (2019). Important parameters in plasma jets for the production of RONS in liquids for plasma medicine: A brief review. Front. Chem. Sci. Eng..

[33] Wende K (2015). Identification of the biologically active liquid chemistry induced by a nonthermal atmospheric pressure plasma jet. Biointerphases.

[34] Adamovich I (2017). The 2017 Plasma Roadmap: Low temperature plasma science and technology. J. Phys. D. Appl. Phys..

[35] Ranieri P (2020). GSH modification as a marker for plasma source and biological response comparison to plasma treatment. Appl. Sci..

[36] Privat-Maldonado A (2017). Nontarget biomolecules alter macromolecular changes induced by bactericidal low-temperature plasma. IEEE Trans. Radiat. Plasma Med. Sci..

[37] Labay C (2020). Enhanced generation of reactive species by cold plasma in gelatin solutions for selective cancer cell death. ACS Appl. Mater. Interfaces.

[38] Chung T-H (2020). Cell electropermeabilisation enhancement by non-thermal-plasma-treated PBS. Cancers.

[39] Miller V, Lin A, Fridman A (2016). Why target immune cells for plasma treatment of cancer. Plasma Chem. Plasma Process..

[40] Saito K (2016). Tumor-selective mitochondrial network collapse induced by atmospheric gas plasma-activated medium. Oncotarget.

[41] Adachi T (2015). Plasma-activated medium induces A549 cell injury via a spiral apoptotic cascade involving the mitochondrial-nuclear network. Free Radic. Biol. Med..

[42] Biscop L (2019). Influence of cell type and culture medium on determining cancer selectivity of cold atmospheric plasma treatment. Cancers.

[43] Van Boxem W (2017). Anti-cancer capacity of plasma-treated PBS: Effect of chemical composition on cancer cell cytotoxicity. Sci. Rep..

[44] Bauer G (2019). Cold atmospheric plasma and plasma-activated medium: Antitumor cell effects with inherent synergistic potential. Plasma Med..

[45] Girard P-M (2016). Synergistic effect of H2O2 and NO2 in cell death induced by cold atmospheric He plasma. Sci. Rep..

[46] Nakamura K (2018). Intraperitoneal treatment with plasma-activated liquid inhibits peritoneal metastasis in ovarian cancer mouse model. Clin. Plasma Med..

[47] Sato Y (2018). Effect of plasma-activated lactated Ringer’s solution on pancreatic cancer cells in vitro and in vivo. Ann. Surg. Oncol..

[48] Metelmann H-R (2018). Clinical experience with cold plasma in the treatment of locally advanced head and neck cancer. Clin. Plasma Med..

[49] Tanaka H (2016). Non-thermal atmospheric pressure plasma activates lactate in Ringer’s solution for anti-tumor effects. Sci. Rep..

[50] Yan D, Sherman JH, Keidar M (2017). The application of the cold atmospheric plasma-activated solutions in cancer treatment. Anticancer Agents Med. Chem..

[51] Solé-Martí X, Espona-Noguera A, Ginebra MP, Canal C (2021). Plasma-conditioned liquids as anticancer therapies in vivo: Current state and future directions. Cancers.

[52] Zaplotnik R (2015). Influence of a sample surface on single electrode atmospheric plasma jet parameters. Spectrochim. Acta.

[53] Green LC (1982). Analysis of nitrate, nitrite, and [15N]nitrate in biological fluids. Anal. Biochem..

[54] Giustarini D, Rossi R, Milzani A, Dalle-Donne I (2008). Nitrite and nitrate measurement by Griess reagent in human plasma: Evaluation of interferences and standardization. Methods Enzymol..

[55] Guevara I (1998). Determination of nitrite/nitrate in human biological material by the simple Griess reaction. Clin. Chim. Acta.

[56] Mishin V, Gray JP, Heck DE, Laskin DL, Laskin JD (2010). Application of the Amplex red/horseradish peroxidase assay to measure hydrogen peroxide generation by recombinant microsomal enzymes. Free Radic. Biol. Med..

