# Supplementary material for: Selectivity of direct plasma treatment and plasma-conditioned media in bone cancer cell lines
Source: Sci Rep. 2021 Sep 1;11:17521. doi: 10.1038/s41598-021-96857-9 (PMC8410816; doi:10.1038/s41598-021-96857-9)
Supplement: Supplementary file 1 — Supplementary Information. [file 41598_2021_96857_MOESM1_ESM.docx]

**Supplementary information**

Table S1: Chemical composition of AdvDMEM and Supplemented AdvDMEM cell culture media.

| **Composition (%)** | **AdvDMEM** | **Supplemented AdvDMEM** |
| --- | --- | --- |
| AlbuMax®II | Infinity | Infinity |
| Human Transferrin | Infinity | Infinity |
| Insulin Recombinant Full Chain | Infinity | Infinity |
| Sodium chloride | 95.1 | 95.1 |
| Sodium bicarbonate | 2.3 | 2.3 |
| D-glucose | 1.4 | 1.4 |
| Potassium chloride | 0.3 | 0.3 |
| Calcium chloride | 0.1 | 0.1 |
| Sodium phosphate monobasic | 0.04 | 0.04 |
| Sodium pyruvate | 0.1 | 0.1 |
| FBS | - | 10 |
| L-Glutamine | - | 1 |
| Penicillin/Streptomycin | - | 1 |


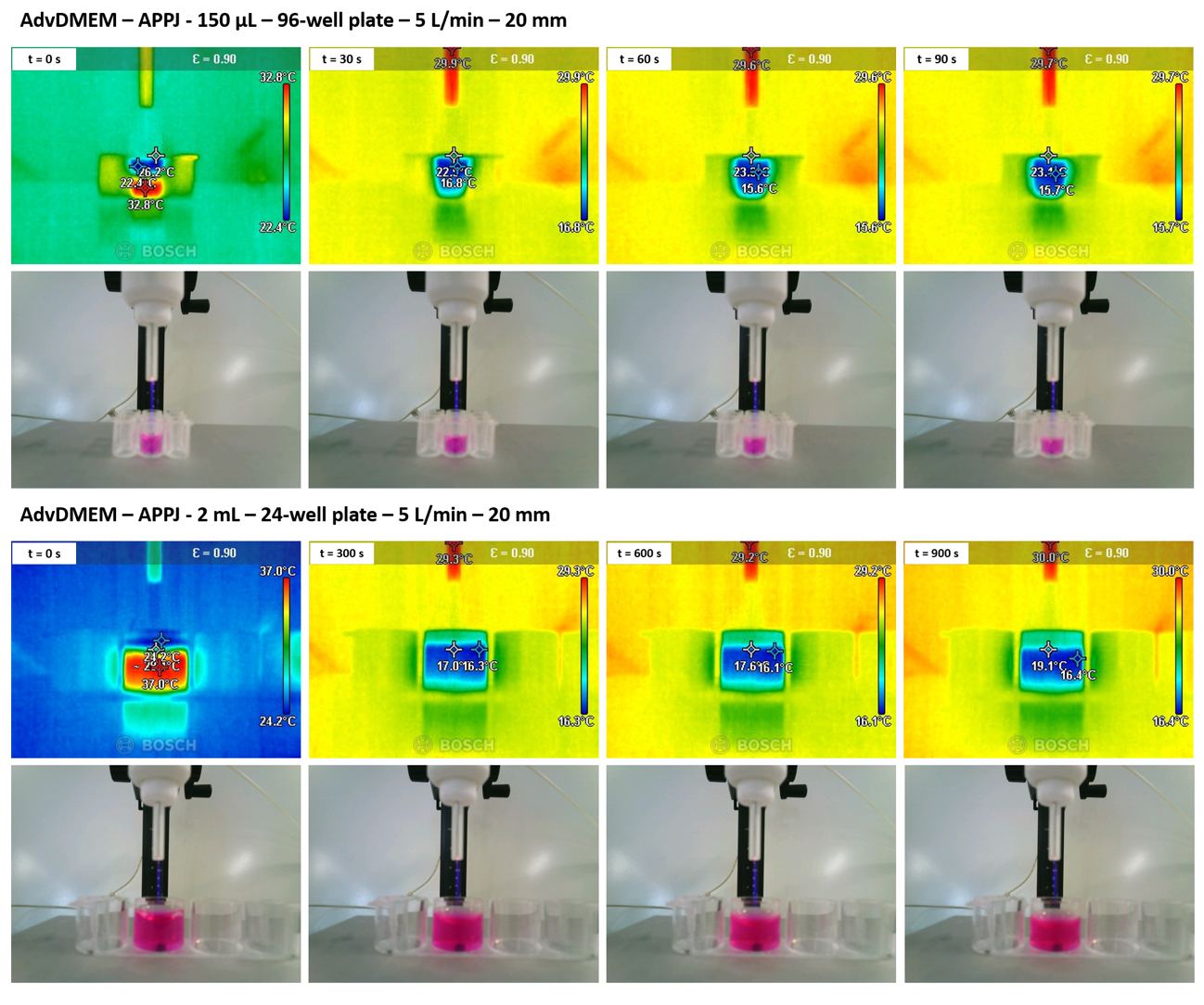


Figure S1: Monitoring of the cell culture media temperature during APPJ treatment for a volume of 150 µL in 96-well plate (top) and for a volume of 2 mL in 24-well plate (bottom).
